# Supplementary material for: Identification and prediction of Parkinson’s disease subtypes and progression using machine learning in two cohorts
Source: NPJ Parkinsons Dis. 2022 Dec 16;8:172. doi: 10.1038/s41531-022-00439-z (PMC9758217; doi:10.1038/s41531-022-00439-z)
Supplement: Supplementary file 1 — Supplementary Information [file 41531_2022_439_MOESM1_ESM.pdf]

Table of Contents

Supplementary Material **4**

Interpretation of the Latent Space 4

Five Year Progression Space 5

Additional Details on Replication Cohort 6

Feature Importance 6

Change in diagnosis status 7

Association testing of Nfl with PD subtypes 7

Supplementary Figures **9**

Supplementary Figure 1. Shows how each 122 different input parameters have been projected into the new dimension of the Parkinson's progression space (cognitive, motor, and sleep dimensions). Darker colors represent strong mapping. The mapping is shown at the final visit after z-score normalization; it is similar for other visits as well (Figure not shown). 9

Supplementary Figure 2. Visualization of two-dimensional progression space of PD subtypes at the end of every year, showing their normalized trajectory. (BL-baseline, V04-Year1, V06-Year2, V08-Year3, V10-Year4, V12-Year5) 10

Supplementary Figure 3. Shows the distribution of projected dimensions (cognitive, motor, and sleep) weights for each Parkinson's category and healthy control after five years. An increase in values along either direction reflects the increase in the disturbance. PDvec3 has the highest motor and sleep disturbance, as well as the highest cognitive impairment. 11

Supplementary Figure 4. shows the progression of each PD subtype over time for motor, sleep, and cognitive dimension overtime on the preprocessed values. 12

Supplementary Figure 5. Shows the progression of each PD subtype over time. The graphs demonstrate the actual clinical values of each subtype overtime for UPDRS-Part I, Part 2, Part 3, as well as Hopkins Verbal Learning Test, Symbol Digit Modalities Test, Semantic Fluency test, Epworth Sleepiness Scale, State-Trait Anxiety Inventory for Adults, and Geriatric Depression Scale. BL: Baseline. V04: visit number 4 after 12 months. V06: visit number 6 after 24 months. V08: visit number 8 after 36 months. V10: visit number 10 after 48 months. V12: visit number 12 after 60 months. 14

Supplementary Figure 6. Shows the summary of clinical parameters (top 50 features) to the prediction models ordered by their importance. The value indicates the scaled importance of the variables in predicting the PD subtypes. Table lists significantly contributing clinical parameters based on the baseline model and on model using both baseline and year 1 data. 15

Supplementary Figure 7: Clinical features influencing Parkinson's Disease progression class. Panels A and B from left to right. Detailed view of influence of top features for Higher PD progression class i.e. PDvec3 (Left) and lower PD progression class i.e. PDvec1 (Right). Higher value on the horizontal axis represents

higher probability of a PD patient belonging to the PDvec3 class (left) and PDvec1 class (right). 16

Supplementary Figure 8: Shows the top-20 features with their importance score for the classification of different PD progression subtypes with the model that uses a combination of demographics (education, year, sex, race), biospecimen (cerebrospinal fluid, serum Nfl levels), genetics (hg genotype), vital signs (weight, height, blood pressure) and UPDRS measurements. 17

Supplementary Figure 9. Shows the trajectory of two PD patients in the two dimensional progression space whose status has changed from their recruitment category in PPMI cohort. It also shows the average trajectory of PD subtypes and Non-PD subjects. The marker size corresponds to time from baseline. 18

Supplementary Figure 10. Kernel Density Estimation (KDE) analysis of Age and MDS-UPDRS Part III (objective motor symptom examination by a trained neurologist) in PPMI and PDBP cohorts. (a) shows the density of Parkinson's participant's age in the 3-years PPMI, PDBP, and 3-years PDBP datasets, and (b) shows the distribution of Parkinson's participant's MDS-UPDRS Part III at baseline in the 3-years PPMI, PDBP, and 3-years PDBP datasets. The three density functions in both figures are similar showing the validity of statistical replication. 19

Supplementary Figure 11. The workflow of predictive modeling evaluation and hyper-parameter tuning. 20

Supplementary Tables **21**

Supplementary Table 1. Shows the longitudinal changes in serum Nfl levels over 5 years for three subtypes. We used a statistical t-test between aPDvec1 vs. PDvec2 and bPDvec1 vs. PDvec3 to compare the means of slope and serum Nfl levels at different points in time. 21

Supplementary Table 2. Shows the top 20 clinical parameters used to obtain 0.92 AUC scores and their mapped dimension. Refer to Table S3(b) for the scaled importance weights of each feature. 22

Supplementary Table 3. Shows the summary of clinical parameters (top 50 features) to the prediction models ordered by their importance. The value indicates the scaled importance of the variables in predicting the PD subtypes. Table lists significantly contributing clinical parameters based on the baseline model and on model using both baseline and year 1 data. 23

Supplementary Table 4. Summary of clinical parameters with significant contributions to the prediction models. Table lists significantly contributing clinical parameters based on baseline examination tests (BL) or based on baseline with year-1 (BL + Y1) test items (+ indicates if a feature is used in predictive models, and - means the feature is not included). Abbreviations: EPS, Epworth Sleepiness Scale; HVLT, Hopkins Verbal Learning Test; LNS, Letter-Number Sequencing; MDS-UPDRS, Movement Disorder Society Revision of the Unified Parkinson's Disease Rating Scale; MoCA, Montreal Cognitive Assessment; RBDSQ, REM Sleep Behavior Disorder Screening Questionnaire; QUIP, Questionnaire for Impulsive-Compulsive Disorders in Parkinson's Disease; SCOPA-AUT, Assessment of Autonomic Dysfunction; STAI, State-Trait Anxiety Inventory. 24

Supplementary Table 5. Two-sample t-test for quantified replication cohort validation analysis. PPMI vs. PDBP (selected participants with 3 years of data). 26
Supplementary Table 6. Shows the description of PPMI clinical assessment features and their labels. 27

Supplementary Material

Interpretation of the Latent Space

To understand the interpretation of PD progressions space dimensions, **Supplementary Figure 1** shows the mapping guide for how the PPMI's high-dimensional space of 122 different clinical parameters is mapped to the three-dimensional embedding of Parkinson's disease progression space. The features are grouped together to represent coherent skills. The leftmost component in **Supplementary Figure 1** mainly constitutes the questionnaire associated with sleep and mood problems, such as dream, fatigue, anxiety, and depression. The middle component represents questions related to motor skills such as speech, facial expression, tremor, and rigidity. The third component represents questions related to cognitive skills, such as cognitive assessment and verbal learning tests. Therefore, the columns represent the projected three dimensions, i.e., motor, cognitive, and sleep-related trajectories, and the rows are the PPMI clinical parameters. This interpretable mapping is due to the property of NMF to group features showing similar variations in the data. This figure allows us to not only observe the conversion but also the heterogeneity of some clinical parameters, for instance how some of the Epworth Sleepiness Scale parameters reflect both sleep and cognitive disorders, and some reflect both sleep and movement disorders. We also looked at the features that seem to be incorrectly assigned, such as cognition (NP1COG) in the motor, and neurocranial (CN346RSP) in sleep. We find that the responses to these questions show minimal variation across subjects, which might make NMF assign them to any of the components. In comparisons of the eigenvalues within the NMF decomposition, the projected motor dimension was responsible for 63.58% of

the explained variance, followed by the sleep dimension (21.81·%), and cognitive dimension (14.61%).

Five Year Progression Space

Supplementary Figure 2 shows the disease trajectory of different PD subtypes. The progression space shows the gradual and linear change for all the subjects. Furthermore, the progression space tends to stabilize at the end of the third year. In this way, our model can capture patients' nuanced behavior showing their progression along with different skills. It is interesting to observe that a significant decline occurs between the second and third year for the subjects in our analysis. In terms of characteristics of PDs identified subtypes, **Supplementary Figure 3** demonstrated how cognitive, motor and sleep-related symptoms differ within each PDs subtype and in controls. There is a clear trend for increased cognitive, sleep, and motor disturbances after five years in fast progressors compared to the slower progressing subtypes. The slowest progressive subtypes (PDvec1) show a mild decline for motor dimension but less change for sleep and cognitive dimensions. We can observe that the difference in progression rates between controls and fastest progressive subtypes is mainly along the motor dimension followed by sleep and then the cognitive dimension.

Supplementary Figure 4 shows the progression of each PD subtype overtime at baseline and after 12 months, 24, 36, 48 months, and 60 months. To better understand the clinical presentation of the three identified subtypes, **Supplementary Figure 4 and 5** demonstrates the three main projected dimensions (motor, cognitive, and sleep-related disturbances), as well as actual clinical values of each subtype overtime for UPDRS-Part I, Part II, Part III, as well as Hopkins Verbal Learning Test, Symbol Digit Modalities Test, Semantic Fluency test, Epworth Sleepiness Scale, State-Trait Anxiety Inventory for Adults, and Geriatric Depression Scale.

Additional Details on Replication Cohort

Supplementary Figure 10 shows PPMI and PDBP cohorts are similarly distributed; hence, they are suitable for replication and validation. Furthermore, we have performed the two-sample t-test for quantified replication cohort validation analysis (Table 1).

Feature Importance

The predictive model was also analyzed to identify the feature importance in predicting PD subtypes. Feature importance is determined by calculating the relative influence of each variable, which is typically given by information gain/entropy, and how much the variable contributes to the accuracy (**Supplementary Figure 6**). We further scaled each feature's importance between 0 and 1 using min-max normalization. **Supplementary Figure 6** shows the top-50 features identified by our predictive model. We list the top 20 features used as input to obtain 0.92 AUC with an ensemble of machine learning models.

SHAP is an unified approach to explain the output of any supervised machine learning model. It assigns an importance value to every feature based on Shapley values. In addition, it generates the impact of each feature on the model's output i.e. the class probability for classification algorithms. The best performing model among five folds is chosen to calculate the SHAP values. **Supplementary Figure 7 (left)** shows the impact score (SHAP contribution) on the probability of PDvec3 class. We see that a higher *serum_nfl* score corresponds to the increase in the probability of a patient belonging to the PDvec3 class. Similarly, higher scores on other symptomatic features related to hobby, sleep are among the top features that can differentiate between PD_h and other classes. **Supplementary Figure 7 (right)** shows the behavior of the model for PDvec1 class. The top features include sleeping behavior, Hoehn and Yahr stage

score and the posture stability with probability of lower progressive class increases with increase in scores for these features. Younger PD patients at screening are expected to show lower PD progression as compared to the older patients. **Supplementary Figure 8** shows the top 20 features involved in classifying PD progressive subtypes.

Change in diagnosis status

The clinical condition of patients can deteriorate, stay the same, or rarely gets better with time. As the study progresses and more information becomes available about the disease manifestation, the patient's diagnosis will be updated. We looked at cases where their clinical diagnosis were updated in the PPMI study. **Supplementary Figure 9**, shows the trajectory of two patients initially diagnosed as PD in the progression space. We can observe that the patient whose status has changed from PD to dementia has much worse condition along the Cognitive dimension. The other patient whose status changed from PD to multiple system atrophy has shown more decline along the motor dimension.

Association testing of Nfl with PD subtypes

Supplementary Table 1 below details baseline and follow-up differences in Nfl across the predicted progression vector classes. Here we show significant differences between slow and faster progressors not only in the measures of Nfl itself but in the slope of change.

Correcting for relevant parameters using a linear mixed-effects model

In addition to subtype, Nfl measurements might be sensitive to other factors such as sex, height, weight, and age at baseline. We used a linear mixed effects model to test for the association between subtypes and Nfl measures after adjustment. For the mixed effects model, we used subtype as categorical input to the model. The model is as follows:

"nfl_i ~ ” β0*years_from_baseline + β1*WGTKG + β2*HTCM + β3*age_at_baseline + β4*sex + β5*I[subtype_i=PDvec2] + β6*I[subtype_i=PDvec2] * years_from_baseline + β7*I[subtype_i=PDvec3] + β8*I[subtype_i=PDvec3] * years_from_baseline + γ0i + γ1i*years_from_baseline"

where γ0i is the random intercept and γ1i is the random slope term for subject i. β0-β8 are fixed effect parameters that are shared by all subjects. I[.] denotes the indicator function.

Effect size estimate of slope term across time for PDvec3 (β8) is statistically significant (P<0.005), with fixed effects = 1.18 [95%CI: 0.39-1.97].

Supplementary Figures

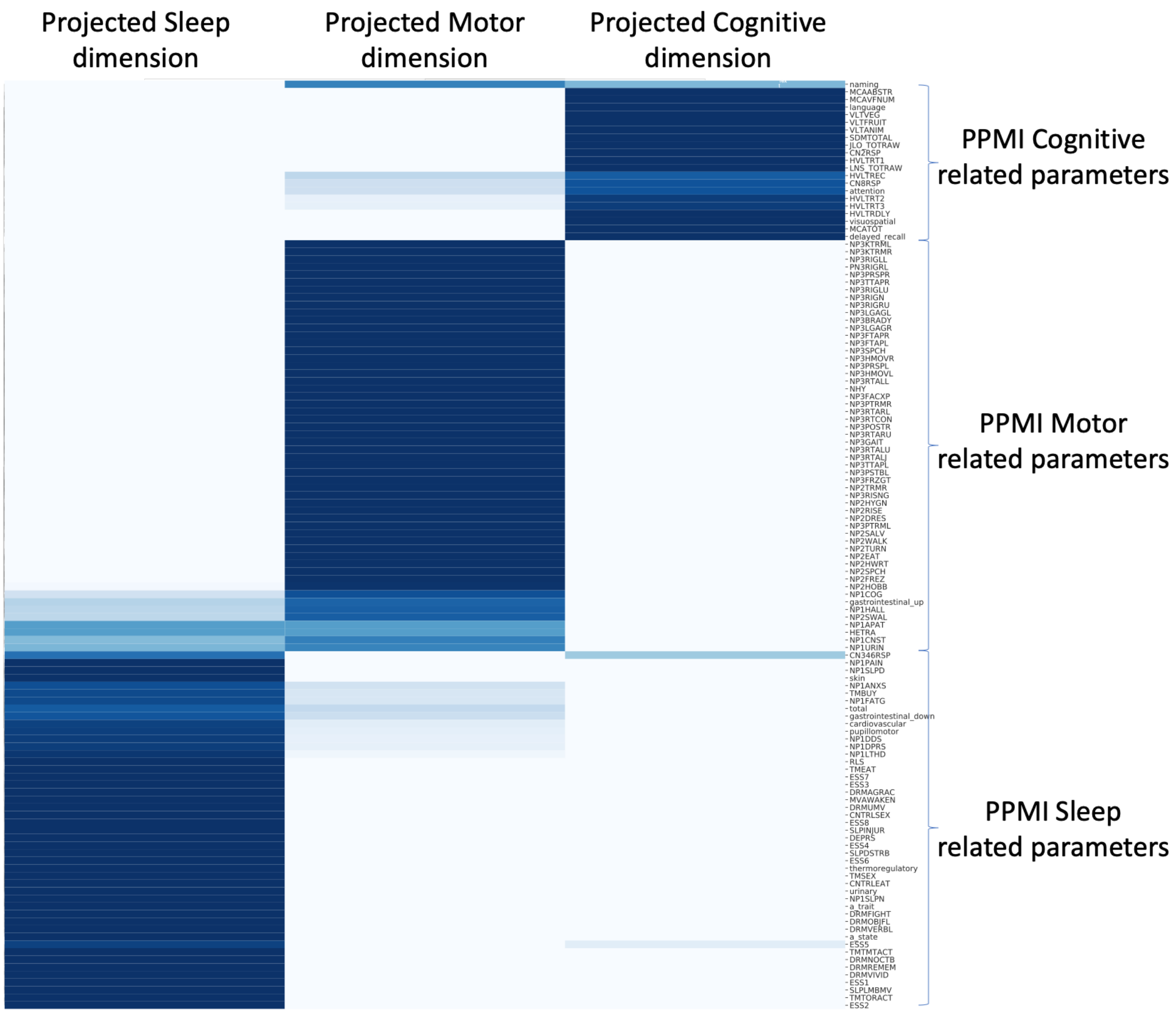

Supplementary Figure 1. Shows how each 122 different input parameters have been projected into the new dimension of the Parkinson's progression space (cognitive, motor, and sleep dimensions). Darker colors represent strong mapping. The mapping is shown at the final visit after z-score normalization; it is similar for other visits as well (Figure not shown).

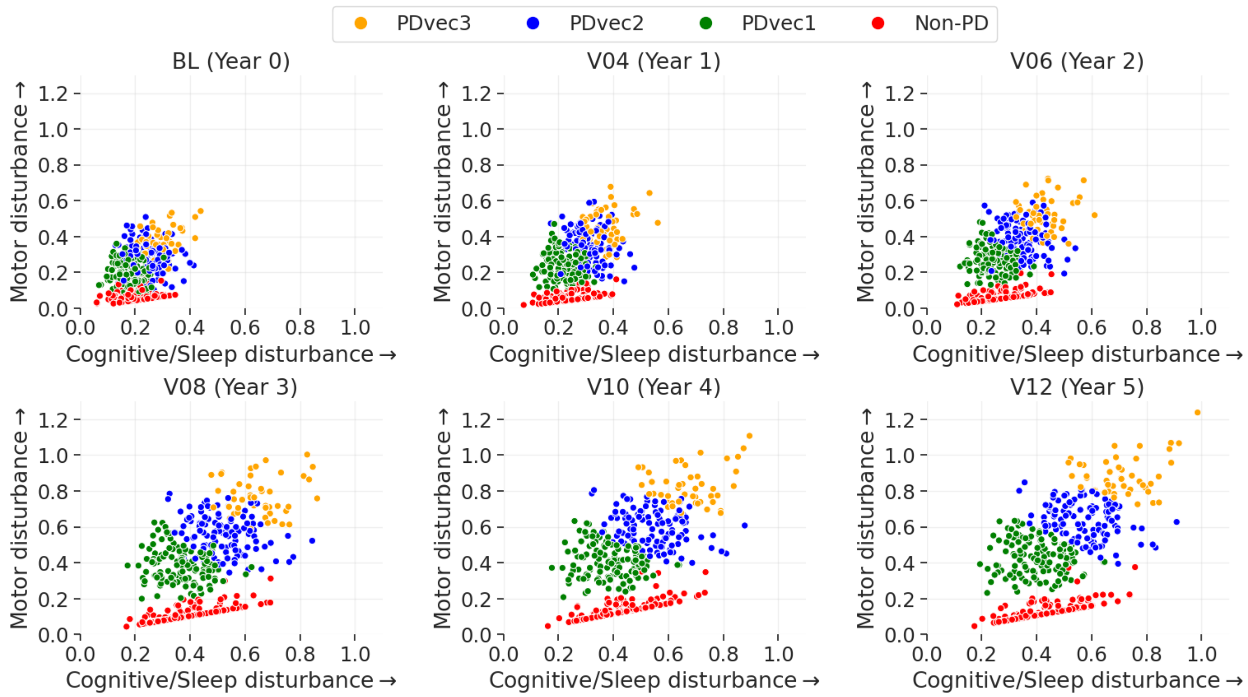

Supplementary Figure 2. Visualization of two-dimensional progression space of PD subtypes at the end of every year, showing their normalized trajectory. (BL-baseline, V04-Year1, V06-Year2, V08-Year3, V10-Year4, V12-Year5)

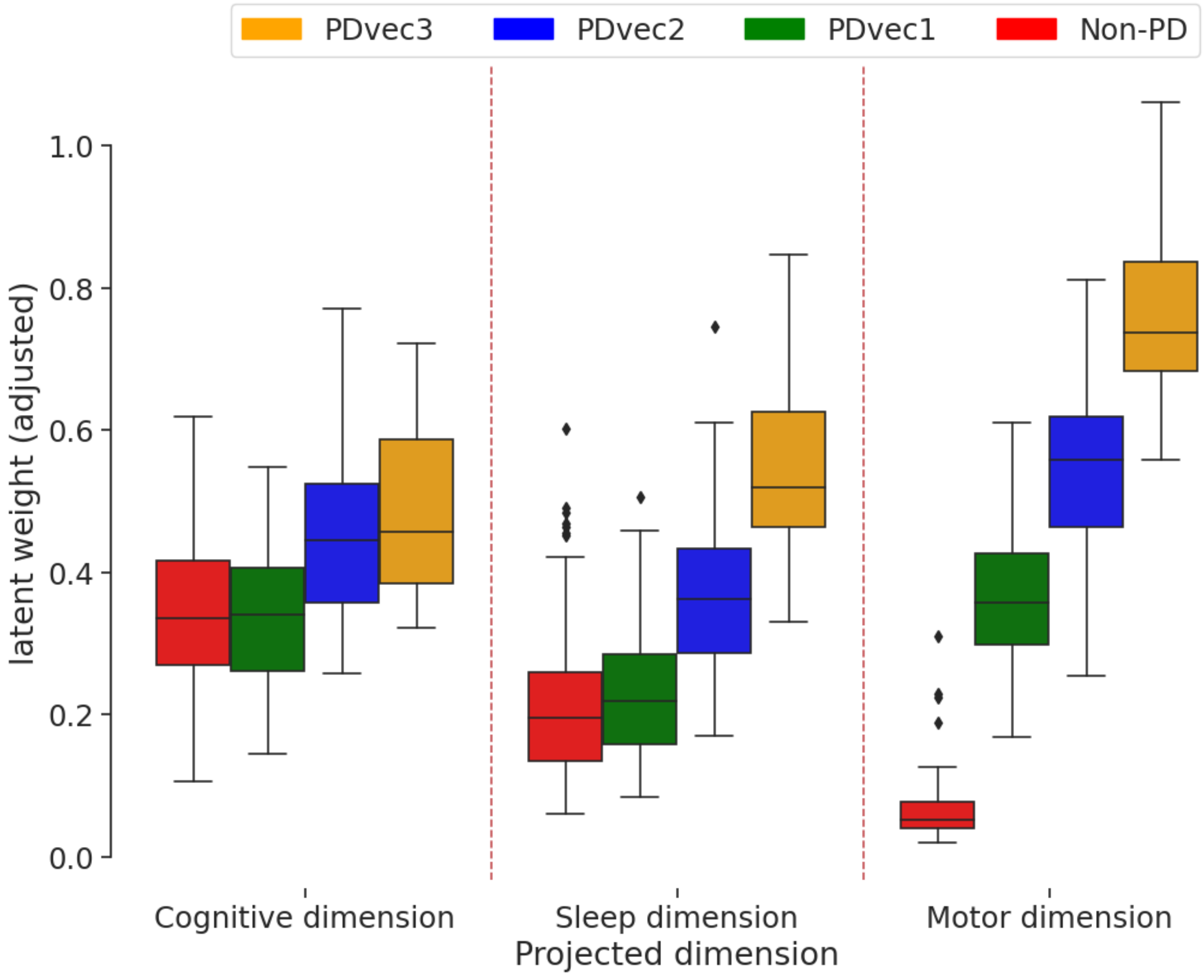

Supplementary Figure 3. Shows the distribution of projected dimensions (cognitive, motor, and sleep) weights for each Parkinson's category and healthy control after five years. An increase in values along either direction reflects the increase in the disturbance. PDvec3 has the highest motor and sleep disturbance, as well as the highest cognitive impairment.

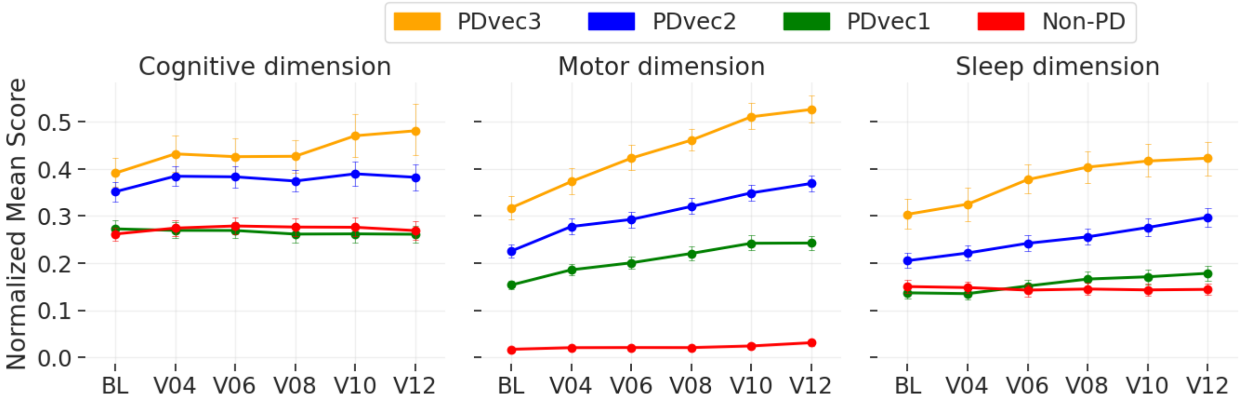

Supplementary Figure 4. shows the progression of each PD subtype over time for motor, sleep, and cognitive dimension overtime on the preprocessed values.

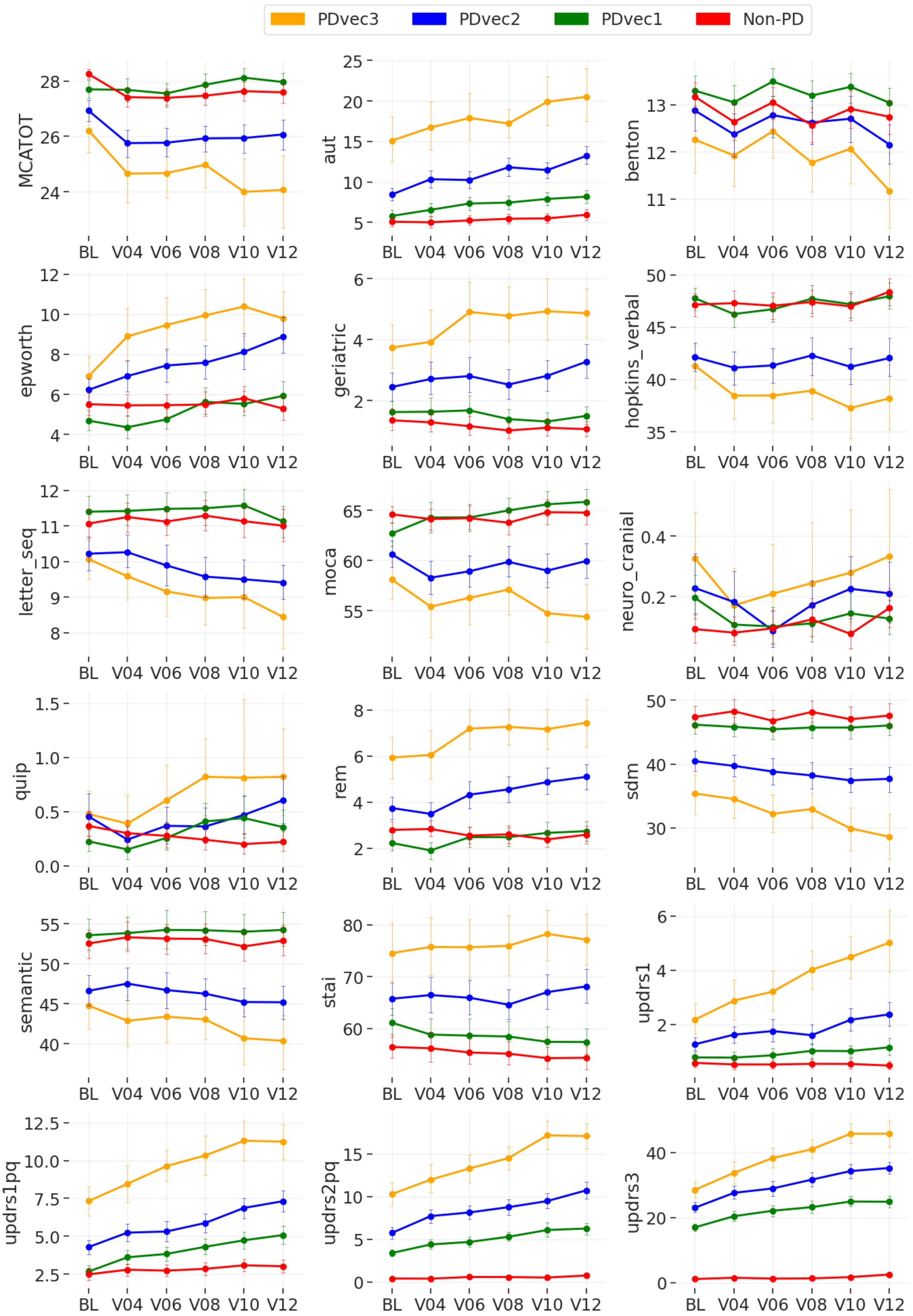
PDvec3
PDvec2
PDvec1
Non-PD
MCATOT
aut
benton
epworth
geriatric
hopkins_verbal
letter_seq
moca
neuro_cranial
quip
rem
sdm
semantic
stai
updrs1
updrs1pq
updrs2pq
updrs3
BL
V04
V06
V08
V10
V12

Supplementary Figure 5. Shows the progression of each PD subtype over time. The graphs demonstrate the actual clinical values of each subtype overtime for UPDRS-Part I, Part 2, Part 3, as well as Hopkins Verbal Learning Test, Symbol Digit Modalities Test, Semantic Fluency test, Epworth Sleepiness Scale, State-Trait Anxiety Inventory for Adults, and Geriatric Depression Scale. BL: Baseline. V04: visit number 4 after 12 months. V06: visit number 6 after 24 months. V08: visit number 8 after 36 months. V10: visit number 10 after 48 months. V12: visit number 12 after 60 months.

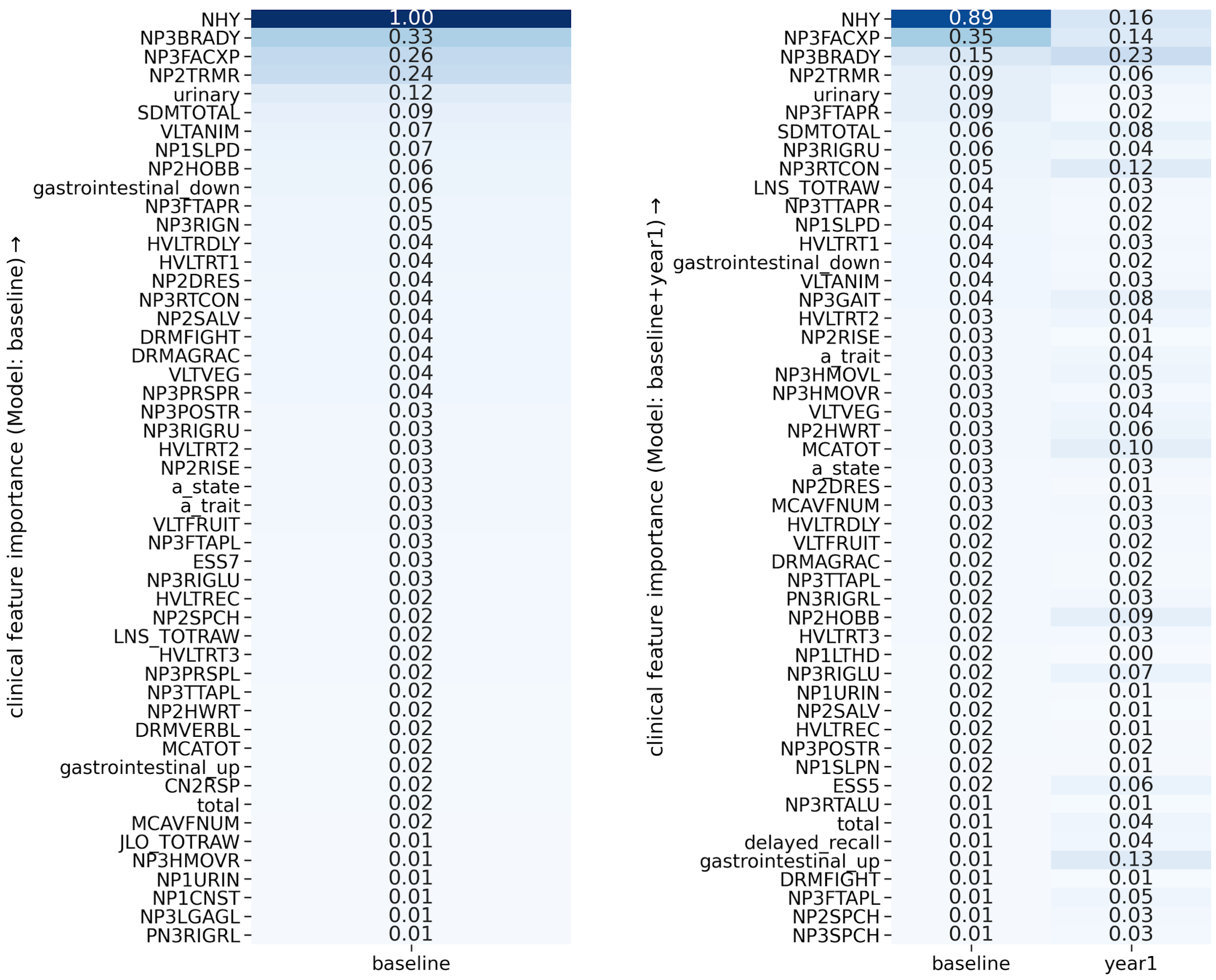

Supplementary Figure 6. Shows the summary of clinical parameters (top 50 features) to the prediction models ordered by their importance. The value indicates the scaled importance of the variables in predicting the PD subtypes. Table lists significantly contributing clinical parameters based on the baseline model and on model using both baseline and year 1 data.

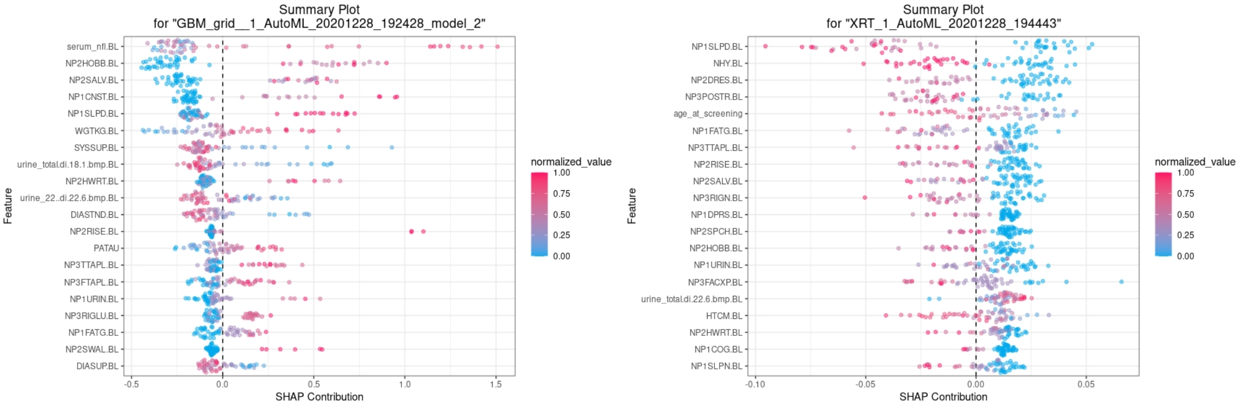

Supplementary Figure 7: Clinical features influencing Parkinson's Disease progression class. Panels A and B from left to right. Detailed view of influence of top features for Higher PD progression class i.e. PDvec3 (Left) and lower PD progression class i.e. PDvec1 (Right). Higher value on the horizontal axis represents higher probability of a PD patient belonging to the PDvec3 class (left) and PDvec1 class (right).

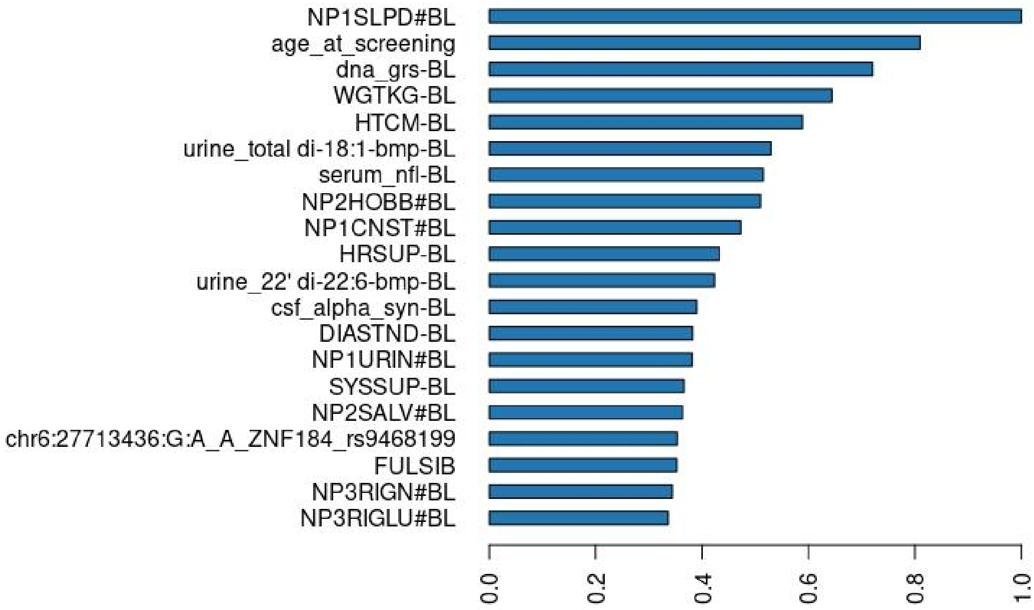

Supplementary Figure 8: Shows the top-20 features with their importance score for the classification of different PD progression subtypes with the model that uses a combination of demographics (education, year, sex, race), biospecimen (cerebrospinal fluid, serum Nfl levels), genetics (hg genotype), vital signs (weight, height, blood pressure) and UPDRS measurements.

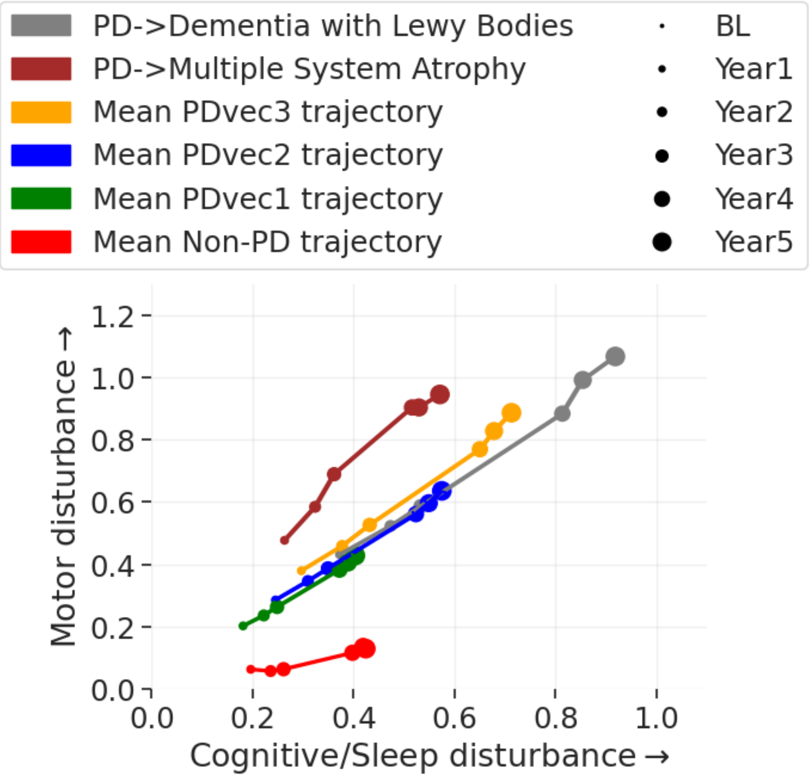

Supplementary Figure 9. Shows the trajectory of two PD patients in the two dimensional progression space whose status has changed from their recruitment category in PPMI cohort. It also shows the average trajectory of PD subtypes and Non-PD subjects. The marker size corresponds to time from baseline.

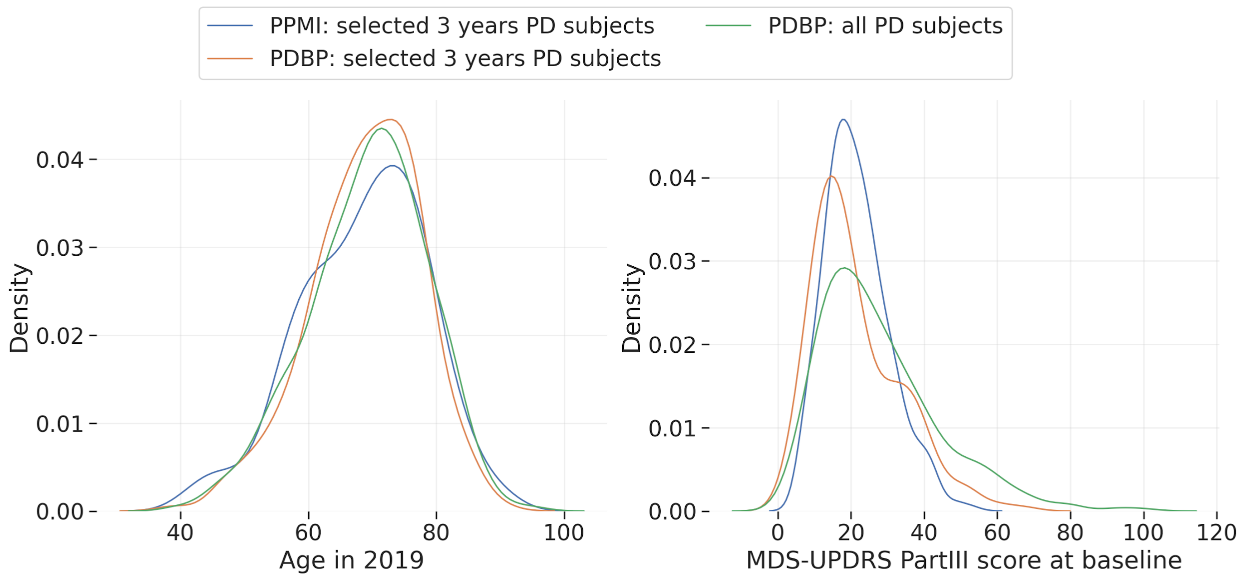

Supplementary Figure 10. Kernel Density Estimation (KDE) analysis of Age and MDS-UPDRS Part III (objective motor symptom examination by a trained neurologist) in PPMI and PDBP cohorts. (a) shows the density of Parkinson's participant's age in the 3-years PPMI, PDBP, and 3-years PDBP datasets, and (b) shows the distribution of Parkinson's participant's MDS-UPDRS Part III at baseline in the 3-years PPMI, PDBP, and 3-years PDBP datasets. The three density functions in both figures are similar showing the validity of statistical replication.

```
Algorithm 1: Model Evaluation Procedure
for each iteration i=1,2,..I do
    Divide the dataset into train (80%) and test data (20%) at random;
    Divide the train into K cross-validation subfolds at random;
    for each fold k=1,2,..K // CV loop do
        validation = fold k;
        subtrain = all folds other than k;
        Train model with every hyperparameter on subtrain;
        Evaluate it on validation;
    end
    Calculate the average metrics score on validation over the K folds
      for every hyperparameter;
    Choose the best hyperparameter setting;
    Train a model with the best hyperparameter on train;
    Evaluate its performance on test;
end
Calculate the mean accuracy over all I iterations on test data;
```

Supplementary Figure 11. The workflow of predictive modeling evaluation and hyper-parameter tuning.

Supplementary Tables

Supplementary Table 1. Shows the longitudinal changes in serum Nfl levels over 5 years for three subtypes. We used a statistical t-test between [a]PDvec1 vs. PDvec2 and [b]PDvec1 vs. PDvec3 to compare the means of slope and serum Nfl levels at different points in time.

**Outcome**	**PDvec1**	**PDvec2**	**PDvec3**
Δ serum Nfl level per year Mean [SD]	1.31 [2.36]	1.48 [2.76]	2.91 [4.23]
t-test P-value [t-statistic]	-	0.6196[a] [-0.50]	0.0025[b] [-3.07]
Baseline Mean [SD]	11.54 [5.84]	11.77 [5.21]	15.42 [6.66]
t-test P-value [t-statistic]	-	0.7576[a] [-0.31]	0.0004[b] [-3.63]
At end of year1 Mean [SD]	11.72 [5.05]	13.69 [10.42]	15.72 [6.38]
t-test P-value [t-statistic]	-	0.086[a] [-1.73]	0.0002[b] [-3.86]
At end of year2 Mean [SD]	13.34 [8.09]	13.85 [7.17]	17.09 [7.91]
t-test P-value [t-statistic]	-	0.6321[a] [-0.48]	0.0138[b] [-2.49]
At end of year3 Mean [SD]	14.16 [8.98]	14.76 [8.98]	19.87 [8.99]
t-test P-value [t-statistic]	-	0.5761[a] [-0.56]	0.0006[b] [-3.51]
At end of year5 Mean [SD]	16.72 [13.51]	18.02 [12.36]	26.75 [20.41]
t-test P-value [t-statistic]	-	0.4435[a] [-0.77]	0.0003[b] [-3.73]

Supplementary Table 2. Shows the top 20 clinical parameters used to obtain 0.92 AUC scores and their mapped dimension. Refer to Table S3(b) for the scaled importance weights of each feature.

**Feature**	**Description**	**Latent dimension**
NHY	Hoehn and Yahr stage	Motor
NP3BRADY	Global Spontaneity of movement	Motor
NP3FACXP	Facial expression	Motor
NP2TRMR	Tremor	Motor
urinary	difficulty retaining urine + involuntary loss of urine + stream of urine been weak + pass urine at night + urine your bladder was not completely empty + urine again within 2 hours of the previous time	Sleep
SDMTOTAL	total symbol digit modalities test	Cognitive
VLTANIM	Total number of animals	Cognitive
NP1SLPD	Daytime sleepiness	Sleep
NP2HOBB	Doing hobbies and other activities	Motor
gastrointestinal_down	Have feeling during meal that you were full very quickly + Had problems with constipation + Had to strain hard to press stools + Had involuntary loss of stools	Sleep
NP3FTAPR	Finger tapping right hand	Motor
NP3RIGN	Rigidity – neck	Motor
HVTRT1	Immediate Recall Trial 1	Cognitive
NP2DRES	Dressing	Motor
NP3RTCON	Constancy of rest	Motor
NP2SALV	Saliva and drooling	Motor
DRMFIGHT	In my dreams: sudden limb movements	Sleep
DRMAGRAC	Dreams frequently have aggressive or action-packed content	Sleep
VLTVEG	Total number of vegetables	Cognitive
NP3PRSPR	Pronation-supination – right hand	Motor

Supplementary Table 3. Shows the summary of clinical parameters (top 50 features) to the prediction models ordered by their importance. The value indicates the scaled importance of the variables in predicting the PD subtypes. Table lists significantly contributing clinical parameters based on the baseline model and on model using both baseline and year 1 data.

clinical feature importance (Model: baseline) →

	baseline
NHY	1.00
NP3BRADY	0.33
NP3FACXP	0.26
NP2TRMR	0.24
urinary	0.12
SDMTOTAL	0.09
VLTANIM	0.07
NP1SLPD	0.07
NP2HOBB	0.06
gastrointestinal_down	0.06
NP3FTAPR	0.05
NP3RIGN	0.05
HVLTRDLY	0.04
HVLTRT1	0.04
NP2DRES	0.04
NP3RTCON	0.04
NP2SALV	0.04
DRMFIGHT	0.04
DRMAGRAC	0.04
VLTVEG	0.04
NP3PRSPR	0.04
NP3POSTR	0.03
NP3RIGRU	0.03
HVLTRT2	0.03
NP2RISE	0.03
a_state	0.03
a_trait	0.03
VLTFRUIT	0.03
NP3FTAPL	0.03
ESS7	0.03
NP3RIGLU	0.03
HVLTREC	0.02
NP2SPCH	0.02
LNS_TOTRAW	0.02
HVLTRT3	0.02
NP3PRSPL	0.02
NP3TTAPL	0.02
NP2HWRT	0.02
DRMVERBL	0.02
MCATOT	0.02
gastrointestinal_up	0.02
CN2RSP	0.02
total	0.02
MCAVFNUM	0.02
JLO_TOTRAW	0.01
NP3HMOVR	0.01
NP1URIN	0.01
NP1CNST	0.01
NP3LGAGL	0.01
PN3RIGRL	0.01

clinical feature importance (Model: baseline+year1) →

	baseline	year1
NHY	0.89	0.16
NP3FACXP	0.35	0.14
NP3BRADY	0.15	0.23
NP2TRMR	0.09	0.06
urinary	0.09	0.03
NP3FTAPR	0.09	0.02
SDMTOTAL	0.06	0.08
NP3RIGRU	0.06	0.04
NP3RTCON	0.05	0.12
LNS_TOTRAW	0.04	0.03
NP3TTAPR	0.04	0.02
NP1SLPD	0.04	0.02
HVLTRT1	0.04	0.03
gastrointestinal_down	0.04	0.02
VLTANIM	0.04	0.03
NP3GAIT	0.04	0.08
HVLTRT2	0.03	0.04
NP2RISE	0.03	0.01
a_trait	0.03	0.04
NP3HMOVL	0.03	0.05
NP3HMOVR	0.03	0.03
VLTVEG	0.03	0.04
NP2HWRT	0.03	0.06
MCATOT	0.03	0.10
a_state	0.03	0.03
NP2DRES	0.03	0.01
MCAVFNUM	0.03	0.03
HVLTRDLY	0.02	0.03
VLTFRUIT	0.02	0.02
DRMAGRAC	0.02	0.02
NP3TTAPL	0.02	0.02
PN3RIGRL	0.02	0.03
NP2HOBB	0.02	0.09
HVLTRT3	0.02	0.03
NP1LTHD	0.02	0.00
NP3RIGLU	0.02	0.07
NP1URIN	0.02	0.01
NP2SALV	0.02	0.01
HVLTREC	0.02	0.01
NP3POSTR	0.02	0.02
NP1SLPN	0.02	0.01
ESS5	0.02	0.06
NP3RTALU	0.01	0.01
total	0.01	0.04
delayed_recall	0.01	0.04
gastrointestinal_up	0.01	0.13
DRMFIGHT	0.01	0.01
NP3FTAPL	0.01	0.05
NP2SPCH	0.01	0.03
NP3SPCH	0.01	0.03

Supplementary Table 4. Summary of clinical parameters with significant contributions to the prediction models. Table lists significantly contributing clinical parameters based on baseline examination tests (BL) or based on baseline with year-1 (BL + Y1) test items (+ indicates if a feature is used in predictive models, and - means the feature is not included). Abbreviations: EPS, Epworth Sleepiness Scale; HVLT, Hopkins Verbal Learning Test; LNS, Letter-Number Sequencing; MDS- UPDRS, Movement Disorder Society Revision of the Unified Parkinson's Disease Rating Scale; MoCA, Montreal Cognitive Assessment; RBDSQ, REM Sleep Behavior Disorder Screening Questionnaire; QUIP, Questionnaire for Impulsive-Compulsive Disorders in Parkinson's Disease; SCOPA-AUT, Assessment of Autonomic Dysfunction; STAI, State-Trait Anxiety Inventory.

Clinical Scales	Model	
Specific Test Item(s) (*Parameter Name*)	BL	BL+Y1
**MDS-UPDRS Part 1**		
1.7 Sleep problems (*NP1SLPN*)	+	+
1.8 Daytime Sleepiness (*NP1SLPD*)	+	+
1.10 Urinary Problems (*NP1URN*)	+	+
1.11 Constipation problems (*NP1CNST*)	+	-
1.12 Lightheadedness on Standing (*NP1LTHD*)	-	+
**MDS-UPDRS Part 2**		
2.1 Speech (*NP2SPCH*)	+	+
2.2 Saliva and drooling (*NP2SALV*)	+	+
2.5 Dressing (*NP2DRES*)	+	+
2.7 Handwriting (*NP2HWRT*)	+	+
2.8 Doing hobbies and other activities (*NP2HOBB*)	+	+
2.10 Tremor (*NP2TRMR*)	+	+
2.11 Getting out of bed, car, deep chair (*NP2RISE*)	+	+
**MDS-UPDRS Part 3**		
3.1 Speech (*NP3SPCH*)	-	+
3.2 Facial expression (*NP3FACXP*)	+	+
3.3a Rigidity – neck (*NP3RIGN*)	+	-
3.3b Rigidity – RUE (*NP3RIGRU*)	+	+
3.3c Rigidity – LUE (*NP3RIGLU*)	+	+
3.3d Rigidity – RLE (*NP3RIGRL*)	+	+
3.4a Finger tapping right hand (*NP3FTAPR*)	+	+
3.4b Finger Tapping Left Hand (*NP3FTAPL*)	+	+
3.5a Hand movements - Right Hand (*NP3HMOVR*)	+	+
3.5b Hand movements – left hand (*NP3HMOVL*)	-	+
3.6a Pronation-supination – right hand (*NP3PRSPR*)	+	-
3.6b Pronation-supination – left hand (*NP3PRSPL*)	+	-
3.7a Toe tapping - Right foot (*NP3TTAPR*)	-	+
3.7b Toe tapping – left foot (*NP3TTAPL*)	+	+
3.8b Leg agility – left leg (*NP3LGAGL*)	+	-
3.10 Gait (*NP3GAIT*)	-	+
3.13 Posture (*NP3POSTR*)	+	+
3.14 Global Spontaneity of movement (*NP3BRADY*)	+	+
3.17b Rest tremor amplitude- LUE (*NP3RTALU*)	-	+
3.18 Constancy of rest (*NP3RTCON*)	+	+
3.21 Hoehn and Yahr stage (*NHY*)	+	+
**MoCA**		

Verbal fluency (*MCAVFNUM*)	+	+
MoCA total score (*MCATOT*)	+	+
**HVLT**		
Immediate Recall Trial 1 (*HVLTRT1*)	+	+
Immediate Recall Trial 2 (*HVLTRT2*)	+	+
Immediate Recall Trial 3 (*HVLTRT3*)	+	+
Delayed Recall (*HVLTRDLY*)	+	+
Recognition (*HVLTREC*)	+	+
**LNS**		
LNS-Sum questions 1-7 (*LNS_TOTRAW*)	+	+
**BENTON**		
Judgment line of action total raw score (*JLO_TOTRAW*)	+	-
**RBDSQ**		
Dreams frequently have aggressive or action-packed content (*DRMAGRAC*)	+	+
In my dreams: speaking, shouting, swearing (*DRMVERBL*)	+	-
In my dreams: sudden limb movements (*DRMFIGHT*)	+	+
**EPS**		
Lying down to rest in the afternoon (*ESS5*)	-	+
Sitting quietly after a lunch (*ESS7*)	+	-
**SCOPA-AUT**		
Difficulty retaining urine + involuntary loss of urine + stream of urine been weak + pass urine at night + urine your bladder was not completely empty + urine again within 2 hours of the previous time (*urinary*)	+	+
Had difficulty swallowing or have choked + Has saliva dribbled out of your mouth + Has food become stuck in your throat (*gastrointestinal_up*)	+	+
Have feeling during meal that you were full very quickly + Had problems with constipation + Had to strain hard to press stools + Had involuntary loss of stools (*gastrointestinal_down*)	+	+
**Semantic Fluency**		
Total number of animals (*VLTANIM*)	+	+
Total number of vegetables (*VLTVEG*)	+	+
Total number of fruits (*VLTFRUIT*)	+	+
**STAI**		
Anxiety state score (*a_state*)	+	+
Anxiety trait score (*a_trait*)	+	+
**SDM**		
Total symbol digit modalities test (*SDMTOTAL*)	+	+
**Neuro Cranial**		
Abnormality in Cranial Nerves (*CN2RSP*)	+	-
**Geriatric**		
Geriatric depression total score (*total*)	+	+

Supplementary Table 5. Two-sample t-test for quantified replication cohort validation analysis. PPMI vs. PDBP (selected participants with 3 years of data).

**PPMI vs PDBP (after 3 years)**	**t-value (95% CI)**	**p-value (95% CI)**
Age in 2019	-0.41	0.68
MDS UPDRS PartIII	0.29	0.77

Supplementary Table 6. Shows the description of PPMI clinical assessment features and their labels.

**MDS-UPDRS Part 1**	
NP1DPRS	Depressed mood
NP1ANXS	Anxious mood
NP1SLPN	Sleep problems
NP1PAIN	Pain and other sensations
NP1CNST	Constipation problems
NP1FATG	Fatigue
NP1DDS	Dopamine dysregulation syndrome
NP1SLPD	Daytime sleepiness
NP1URIN	Urinary problems
NP1HALL	Hallucinations
NP1APAT	Apathy
NP1COG	Cognitive impairment
NP1LTHD	Lightheadedness on standing
**MDS-UPDRS Part 2**	
NP2SALV	Saliva and drooling
NP2EAT	Eating tasks
NP2DRES	Dressing
NP2HYGN	Hygiene
NP2HWRT	Handwriting
NP2HOBB	Doing hobbies and other activities
NP2RISE	Getting out of bed, car, deep chair
NP2FREZ	Freezing
NP2SWAL	Chewing and swallowing
NP2TURN	Turning in bed
NP2WALK	Walking and balance
NP2SPCH	Speech
NP2TRMR	Tremor
**MDS-UPDRS Part 3**	
NP3SPCH	Speech
NP3FACXP	Facial expression
NP3RIGN	Rigidity – neck
NP3RIGRU	Rigidity – RUE
NP3RIGRL	Rigidity – RLE
NP3RIGLL	Rigidity – LLE
NP3FTAPR	Finger tapping right hand
NP3FTAPL	Finger tapping left hand
NP3HMOVL	Hand movements – left hand
NP3HMOVR	Hand movements – right hand
NP3PRSPR	Pronation-supination – right hand
NP3PRSPL	Pronation-supination – left hand
NP3TTAPL	Toe tapping – left foot
NP3TTAPR	Toe tapping – right foot
NP3LGAGR	Leg agility – right leg
NP3LGAGL	Leg agility – left leg
NP3RISNG	Arising from chair

NP3POSTR	Posture
NP3BRADY	Global Spontaneity of movement
NP3KTRML	Kinetic tremor – left hand
NP3RTARL	Rest tremor amplitude – RLE
NP3RTALL	Rest tremor amplitude – LLE
NHY	Hoehn and Yahr stage
DYSKPRES	Presence of dyskinesias
NP3FRZGT	Freezing of gait
NP3RTALJ	Rest tremor amplitude - Lip/jaw
NP3RTCON	Constancy of rest
NP3GAIT	Gait
NP3RTALU	Rest tremor amplitude - LUE
NP3RTARU	Rest tremor amplitude - RUE
NP3PTRMR	Pronation - Supination Movements - Right Hand
NP3PTRML	Pronation - Supination Movements - Left Hand
NP3PSTBL	Postural stability
**MoCA**	
Naming	Naming total score
Language	Language total score
Delayed recall	Delayed recall total score
visuospatial	Visuospatial total score
attention	Attention total score
MCAVFNUM	Verbal fluency
MCAABSTR	Abstraction
MCATOT	MoCA total score
**HVLT**	
HVLTRT1	Immediate Recall Trial 1
HVLTRT3	Immediate Recall Trial 3
HVLTRT2	Immediate Recall Trial 2
HVLTRDLY	Delayed Recall
HVLTREC	Recognition
HVLTFPRL	Recognition – false positives, related
**LNS**	
LNS_TOTRAW	LNS-Sum questions 1-7
**QUIP**	
TMSEX	Think having too much sex behavior
TMGAMBLE	Think having too much gambling behavior
TMTORACT	Too much time on recreational activities
CNTRLBUY	Difficulty controlling your buy behaviors
TMTMTACT	Too much time on motor activities
TMTRWD	Too much time on walking/driving activities
TMEAT	Think having too much eating behavior
CNTRLGMB	Difficulty controlling your gambling behaviors
CNTRLSEX	Difficulty controlling your sex behaviors
TMBUY	Think having too much buying behavior
CNTRLEAT	Difficulty controlling your eat behaviors

**RBDSQ**	
DRMAGRAC	Dreams frequently have aggressive or action-packed content
SLPINJUR	I (almost) hurt my bed partner or myself
DRMVERBL	In my dreams: speaking, shouting, swearing
DRMUMV	In my dreams: gestures, complex movements useless during sleep
DRMOBJFL	In my dreams: things fell down around the bed
MVAWAKEN	It happens that my movements awake me
SLPDSTRB	My sleep is frequently disturbed
SLPLMBMV	Know my arms and legs move when asleep
RLS	had RLS
STROKE	Disease of nervous system: stroke
DEPRS	Disease of nervous system: depression
NARCLPSY	had narcolepsy
DRMREMEM	remember the content of my dreams well
DRMNOCTB	dream contents mostly match my nocturnal behaviour
BRNINFM	had inflammatory disease of the brain
DRMVIVID	sometimes have very vivid dreams
HETRA	had head trauma
EPILEPSY	had epilepsy
DRMFIGHT	In my dreams: sudden limb movements
**EPS**	
ESS2	Doze off or fall asleep while watching TV
ESS1	Sitting and reading
ESS3	Sitting, inactive in a public place
ESS4	As a passenger in a car for an hour without a break
ESS5	Lying down to rest in the afternoon
ESS6	Sitting and talking to someone
ESS7	Sitting quietly after a lunch
ESS8	In a car, while stopped for a few minutes
**SCOPA-AUT**	
Gastrointestinal upper	Had difficulty swallowing or have choked + Has saliva dribbled out of your mouth + Has food become stuck in your throat
Gastrointestinal lower	Have feeling during meal that you were full very quickly + Had problems with constipation + Had to strain hard to press stools + Had involuntary loss of stools
thermoregulatory	trouble tolerating cold + trouble tolerating hot
pupillomotor	eyes ever been over-sensitive to bright light
skin	perspired excessively during the day + during the night
cardiovascular	feeling of either becoming light-headed + light-headed after standing for some time + fainted in the past 6 months
urinary	difficulty retaining urine + involuntary loss of urine + stream of urine been weak + pass urine at night + urine your bladder was not completely empty + urine again within 2 hours of the previous time
**Semantic Fluency**	
VLTANIM	Total number of animals
VLTVEG	Total number of vegetables
VLTFRUIT	Total number of fruits

**STAI**	
a_state	Anxiety state score
a_trait	Anxiety trait score
**BENTON**	
JLO_TOTRAW	judgment line of action total raw score
**Geriatric**	
total	geriatric depression total score
**Neuro Cranial**	
CN346RSP, CN8RSP, CN7RSP, CN2RSP, CN910RSP, CN12RSP, CN5RSP, CN11RSP	abnormality in Cranial Nerves
**SDM**	
SDMTOTAL	total symbol digit modalities test
