# Supplementary material for: Identification and prediction of Parkinson’s disease subtypes and progression using machine learning in two cohorts
Source: NPJ Parkinsons Dis. 2022 Dec 16;8:172. doi: 10.1038/s41531-022-00439-z (PMC9758217; doi:10.1038/s41531-022-00439-z)
Supplement: Supplementary file 2 — Reporting Summary [file 41531_2022_439_MOESM2_ESM.pdf]

nature portfolio

Corresponding author(s): Faraz Faghri

Last updated by author(s): Oct 24, 2022

Reporting Summary

Nature Portfolio wishes to improve the reproducibility of the work that we publish. This form provides structure for consistency and transparency in reporting. For further information on Nature Portfolio policies, see our Editorial Policies and the Editorial Policy Checklist.

Statistics

For all statistical analyses, confirm that the following items are present in the figure legend, table legend, main text, or Methods section.

n/a	Confirmed	
☐	☒	The exact sample size (*n*) for each experimental group/condition, given as a discrete number and unit of measurement
☐	☒	A statement on whether measurements were taken from distinct samples or whether the same sample was measured repeatedly
☐	☒	The statistical test(s) used AND whether they are one- or two-sided<br>*Only common tests should be described solely by name; describe more complex techniques in the Methods section.*
☐	☒	A description of all covariates tested
☐	☒	A description of any assumptions or corrections, such as tests of normality and adjustment for multiple comparisons
☐	☒	A full description of the statistical parameters including central tendency (e.g. means) or other basic estimates (e.g. regression coefficient) AND variation (e.g. standard deviation) or associated estimates of uncertainty (e.g. confidence intervals)
☐	☒	For null hypothesis testing, the test statistic (e.g. *F*, *t*, *r*) with confidence intervals, effect sizes, degrees of freedom and *P* value noted<br>*Give P values as exact values whenever suitable.*
☐	☒	For Bayesian analysis, information on the choice of priors and Markov chain Monte Carlo settings
☐	☒	For hierarchical and complex designs, identification of the appropriate level for tests and full reporting of outcomes
☐	☒	Estimates of effect sizes (e.g. Cohen's *d*, Pearson's *r*), indicating how they were calculated

Our web collection on statistics for biologists contains articles on many of the points above.

Software and code

Policy information about availability of computer code

Data collection	The data used in this study was access-controlled from the Parkinson's Progression Marker Initiative (PPMI, http://www.ppmi-info.org/) and the Parkinson’s Disease Biomarkers Program (PDBP, https://pdbp.ninds.nih.gov/), and require individual sign-up to access the data.
Data analysis	The data analysis pipeline for this work was performed in Python (version 3.8) with the support of several open-source libraries (NumPy, pandas, matplotlib, seaborn, plotly, scikit-learn, UMAP, XGBoost, LightGBM, H2O, streamlit). To facilitate replication and expansion of our work, we have made the notebook publicly available on GitHub at https://github.com/anant-dadu/PDProgressionSubtypes.

For manuscripts utilizing custom algorithms or software that are central to the research but not yet described in published literature, software must be made available to editors and reviewers. We strongly encourage code deposition in a community repository (e.g. GitHub). See the Nature Portfolio guidelines for submitting code & software for further information.

Data

Policy information about availability of data

All manuscripts must include a data availability statement. This statement should provide the following information, where applicable:

- Accession codes, unique identifiers, or web links for publicly available datasets
- A description of any restrictions on data availability
- For clinical datasets or third party data, please ensure that the statement adheres to our policy

The data used in this study was access-controlled from the Parkinson's Progression Marker Initiative (PPMI, http://www.ppmi-info.org/) and the Parkinson’s Disease

Biomarkers Program (PDBP, https://pdbp.ninds.nih.gov/), and require individual sign-up to access the data. Additionally, we have developed an interactive website [https://anant-dadu-pdprogressionsubtypes-streamlit-app-aaah95.streamlitapp.com/] where researchers can investigate components of the predictive model and can investigate feature effects on a sample and cohort level.

Human research participants

Policy information about studies involving human research participants and Sex and Gender in Research.

Reporting on sex and gender	Study protocol and manuals are available at the PPMI (www.ppmi-info.org/study-design) and the PDBP (https://pdbp.ninds.nih.gov/) website. This study focuses on identifying data-driven subtypes of Parkinson's disease, irrespective of the sex of an individual. Therefore, sex-specific analysis was not conducted as part of this work.
Population characteristics	Analysis was performed in the PPMI and PDBP datasets, and participant characteristics are outlined in the manuscripts.
Recruitment	Analysis was performed in the PPMI and PDBP datasets, and recruitment criteria is outlined in the manuscripts.
Ethics oversight	The study protocol has been approved by the Parkinson's Progression Markers Initiative (PPMI) database and the Parkinson's Disease Biomarkers Program (PDBP) Consortium.

Note that full information on the approval of the study protocol must also be provided in the manuscript.

Field-specific reporting

Please select the one below that is the best fit for your research. If you are not sure, read the appropriate sections before making your selection.

☒ Life sciences ☐ Behavioural & social sciences ☐ Ecological, evolutionary & environmental sciences

For a reference copy of the document with all sections, see nature.com/documents/nr-reporting-summary-flat.pdf

Life sciences study design

All studies must disclose on these points even when the disclosure is negative.

Sample size	PPMI (n=448); PDBP (n=378)
Data exclusions	Initially misdiagnosed patients and patients with longitudinal data availability of fewer than five years of longitudinal data from PPMI and three years for the PDBP cohort are excluded.
Replication	Validation was performed on PDBP cohort.
Randomization	Separate studies (PPMI; PDBP) under differential enrollment strategies are used for training and validation (more enrollment information is in the manuscript).
Blinding	Blinding was not possible due to the public nature of the data resources used for this study.

Reporting for specific materials, systems and methods

We require information from authors about some types of materials, experimental systems and methods used in many studies. Here, indicate whether each material, system or method listed is relevant to your study. If you are not sure if a list item applies to your research, read the appropriate section before selecting a response.

Materials & experimental systems

n/a	Involved in the study	
☒	☐	Antibodies
☒	☐	Eukaryotic cell lines
☒	☐	Palaeontology and archaeology
☒	☐	Animals and other organisms
☒	☐	Clinical data
☒	☐	Dual use research of concern

Methods

n/a	Involved in the study	
☒	☐	ChIP-seq
☒	☐	Flow cytometry
☒	☐	MRI-based neuroimaging
